# Attitudes and beliefs regarding umbilical cord clamping among midwives, obstetricians, and neonatologists in Sweden: A national cross-sectional survey

**DOI:** 10.1371/journal.pone.0332745

**Published:** 2025-10-08

**Authors:** Maria Wilander, Katarina Ekelöf, Elisabeth Saether, Denice Berglund, Katarina Patriksson, Jenny Svedenkrans, Heike Rabe, Ola Andersson, Li Thies-Lagergren

**Affiliations:** 1 Department of Clinical Sciences, Pediatrics/Neonatology, Lund University, Lund, Sweden; 2 Department of Pediatrics, Hospital of Halland, Halmstad, Sweden; 3 Department of Medicine, Karolinska Institute, Stockholm, Sweden; 4 Department of Research and Development, Region Halland, Halmstad, Sweden; 5 Department of Women, Children and Adolescents, Ålesund Hospital, Møre and Romsdal Hospital Trust, Ålesund, Norway; 6 Department of Obstetrics and Gynecology, Linköping University Hospital, Linköping, Sweden; 7 Department of Health Sciences, University West, Trollhättan, Sweden; 8 Department of Pediatrics/Neonatology, NU-Hospital Group, Sweden; 9 Department of Clinical Science, Intervention, and Technology (CLINTEC), Division of Pediatrics, Karolinska Institutet, Stockholm, Sweden; 10 Department of Neonatology, Karolinska University Hospital, Stockholm, Sweden; 11 Brighton and Sussex Medical School, University of Sussex, Brighton, United Kingdom; 12 Department of Neonatology, University Hospitals Sussex, Brighton, United Kingdom; 13 Department of Neonatology, Skåne University Hospital, Malmö/Lund, Sweden; 14 Department of Health Sciences, Lund University, Lund, Sweden; Federal University of Rio Grande do Norte: Universidade Federal do Rio Grande do Norte, BRAZIL

## Abstract

**Objective:**

The study aimed to explore attitudes and beliefs among midwives, obstetricians, and neonatologists regarding umbilical cord clamping practices, including scenarios involving neonatal compromise. The study was conducted in the context of exploring the potential for implementing intact cord resuscitation.

**Study design:**

A national cross-sectional survey was administered electronically, using an adaptation of the previously used questionnaire developed by Jelin et al. The survey was conducted among midwives, nursing staff, obstetricians, and pediatricians/neonatologists from September 2022 to August 2023. Results were analyzed and reported using descriptive and inferential statistics.

**Result:**

Of 838 respondents analyzed, 94% reported cord clamping timing being “very or moderately important” for neonatal outcomes, where midwives more frequently chose “very important” compared to physicians (p < 0.001). Midwives commonly preferred an event-based (e.g., cessation of pulsations) approach to cord clamping. In scenarios involving resuscitation, 27% of midwives and 10% of pediatric physicians, preferred an event-based approach to cord clamping, with a significant difference between groups (p = 0.005). Among obstetricians, 28.8% reported considering an event-based approach to cord clamping in elective cesarean sections. In resuscitation scenarios, obstetricians predominantly selected < 30 s as the preferred timing for cord clamping, whereas pediatric physicians were less likely to do so (p < 0.001).

**Conclusion:**

Timing of cord clamping is considered important among respondents. The study reveals a generally positive attitude towards delayed cord clamping among health care professionals in Sweden, with notable variations between professional groups. The interprofessional differences highlight the need for shared guidelines and collaborative training to support the potential implementation of intact cord resuscitation.

## Introduction

Cord clamping (CC) practices, particularly in resuscitation scenarios, remain inconsistent worldwide, despite robust evidence supporting delayed CC [1,2]. There is a lack of consensus regarding the definition of delayed CC and recommendations vary between sources [3].

Leaving the umbilical cord intact after birth allows for a placental transfusion and enhances physiological transition from fetal to neonatal life [4,5]. In preterm neonates, delayed CC reduces blood transfusions and decreases mortality [6,7]. In term neonates, delayed CC for 2–5 min increase iron stores, improve brain myelination and neurodevelopmental skills [8–11]. Neonates requiring resuscitation at birth are often excluded from delayed CC studies due to practical challenges, yet emerging evidence suggests that intact cord resuscitation (ICR) may offer benefits, such as improved oxygenation and higher Apgar scores [12,13]. Large randomized controlled trials (RCTs) – including the ongoing SAVE study **(**NCT04070560), and a recent trial by Knol et al. [14,15] – are investigating ICR. Deferring CC is strongly recommended by multiple professional organizations and is the top message in the 2021 European Resuscitation Council guidelines [16]. Current Swedish national guidelines, published in 2022, recommend waiting with CC until the umbilical cord is white and limp in healthy, vaginally born neonates. For cesarean section, the recommendation is to wait at least one minute and in resuscitation situations to clamp in time to ensure initiation of resuscitation measures within 60 seconds [17].

Available data suggest that CC practices differ widely between settings and countries, and more data is needed to understand the attitudes of cord management [2,18,19]. The variability in implementation may arise from factors such as professional attitudes and clinical environments, which create discrepancies in neonatal care, as noted by Blank et al [20]. In Sweden, maternity care is organized around midwives, who independently manage low-risk births and perform delayed CC, often guided by event-based clinical signs such as cessation of pulsations, or placenta delivery [21]. Obstetricians oversee high-risk labors, manage cesarean sections and, together with neonatologists, lead emergency care of mother and newborn. This division of responsibilities influences CC practices: while midwives’ autonomy facilitates adherence to delayed clamping in uncomplicated deliveries, obstetricians and neonatologists may prioritize earlier clamping to facilitate prompt neonatal care when clinical urgency arises.

As new evidence emerges regarding the benefits of delayed CC and ICR, implementation of this evidence into clinical practice is essential [22]. However, implementing new methods, especially complex ones such as ICR, requires involvement of the entire multidisciplinary team [20]. Acceptability is a key outcome in implementation, as it determines whether new interventions – such as ICR – are adopted and sustained in clinical practice [23]. Healthcare professionals’ attitudes and beliefs strongly influence readiness for change and successful adoption of evidence-based practices. Despite Sweden’s uniquely long clamping times, there is a knowledge gap regarding professional perspectives on CC management. Addressing this gap is critical for developing effective implementation strategies. This study is part of the SAVE-study, a hybrid multicenter trial described by Ekelöf et al, which evaluates both clinical outcomes and implementation factors of the SAVE-method for ICR in Sweden [14]. By surveying multidisciplinary team attitudes toward CC timing, this study aims to support the implementation process of the SAVE-study.

## Method

### Study design, setting and participants

A cross-sectional electronic survey was conducted to assess attitudes and beliefs relevant to the implementation of the SAVE-method. This study forms part of the implementation evaluation associated with the SAVE-study; a national multicenter trial primarily aimed at evaluating neonatal clinical outcomes of the SAVE-method for ICR in Sweden. An adapted version of the questionnaire developed by Jelin et al was used (see S1 Appendix for the full questionnaire) [24]. The development and validation of the survey instruments are described in the protocol article [14]. A separate paper describing the instrument validation (IMPRECC) is in manuscript.

A snowball sampling technique was employed, supplemented by additional recruitment strategies to ensure a diverse group of health care professionals across different specialties and roles within maternal and neonatal care. The questionnaire was initially distributed from September 1^st^, 2022, to units involved in the SAVE-study, and expanded in April 2023 to involve all labor- and neonatal units in Sweden. The survey link was emailed, via institutional addresses, to unit managers and physician medical directors, who were asked to forward it to all staff involved in neonatal resuscitation. Posters with QR codes linking to the survey were also displayed at national perinatal meetings. One reminder email was sent to all unit managers and medical directors approximately three months prior to survey closure August 31^st^, 2023. Participation was voluntary. Due to indirect distribution and snowball sampling the exact number of staff reached is unknown; response rates were estimated by comparing respondents to the number of employees per unit, as reported by unit managers in a separate survey. These estimates should be interpreted cautiously.

### Variables and data measurement

Descriptive background variables included age, sex (female/male/other), profession, years of experience, level of neonatal care at the unit, and healthcare region in Sweden. Survey questions were administered either to all participants or to specific professional subgroups.

#### Perceived importance of cord clamping timing.

Questions involved the respondent’s perceived importance of timing of CC in general, and at different gestational ages. Response options on the importance of CC ranged from 1 to 5, with 1 = “very important”, 4 = “not important at all”, and 5 = I don’t know. Gestational ages were divided into < 28 weeks, 28–31 weeks, 32–36 weeks and > 36 weeks. These questions were administered to all respondents.

#### Opinion on cord clamping time.

The questions of health care professionals’ opinions on optimal CC timing for term neonates included both vaginal births and cesarean sections, accounting for the varying clinical scenarios in each context.

Alternatives for CC timing were categorized into time-based intervals (< 30 s, 30–60 s, 1–3 min, 3–6 min, > 6 min) and into event-based timing (cessation of cord pulsations, and placental delivery). Timing options were analyzed individually and categorized into time-based and event-based approaches for group analysis. In this category, questions were specifically tailored to each profession: midwives were queried about vaginal births, obstetricians about cesarean sections, and neonatologists about high-risk labors. Responses containing multiple answers were excluded from analysis.

#### Level of agreement with statements of timing of umbilical cord clamping.

To assess attitudes concerning delayed CC and umbilical cord milking in the resuscitation situation, respondents were asked to consider given statements. Response options ranged from 1 to 5, with 1 = strongly disagree and 5 = strongly agree. The questions were administered to midwives, obstetricians, and neonatologists.

#### Certain clinical situations affecting cord clamping practice.

Questions assessed the influence of specific clinical scenarios (maternal postpartum hemorrhage, risk of neonatal jaundice, neonatal hypothermia, and risk of delayed resuscitation) on respondents’ cord clamping practices. For these questions, the response options ranged from 1 to 6, with 1 = “makes me very inclined to clamp immediately”, 5 = “makes me very inclined to delay clamping” and 6 = no opinion. The questions were administered to midwives and obstetricians. In addition, midwives answered a separate question on the importance of parental preferences for CC time, with response options ranging from “very important” to “not important at all” and “I don’t know”.

#### Local guidelines.

Questions about local guidelines addressing CC management in vigorous and non-vigorous neonates born vaginally or by cesarean section. Response options on guidelines were: (1) yes, there is a guideline recommending delayed CC, (2) yes, there is a guideline recommending early CC, (3) No, there is no guideline, and (4) I don’t know. Responses that contained multiple answers were excluded from the analysis. The questions were administered to all respondents.

### Statistical analysis

Data were presented as numbers (percentages), or medians (range) as appropriate by distribution. Group comparisons in categorical variables were performed using Pearson’s chi-squared test and Fisher’s exact test. To assess differences in ordinal response distributions between groups, the Kruskal–Wallis test was used. When a significant overall difference was detected, post hoc pairwise comparisons were performed using the Mann-Whitney U test, with p-values adjusted using Bonferroni correction. Responses of “I don’t know” were excluded from the comparative analyses of ordinal data. P-values < 0,05 were considered significant. Data were checked for completeness and consistency prior to analysis. Due to a technical issue, multiple responses were allowed in some single-choice questions; such responses were excluded from analysis for those questions only, due to ambiguity. All data were analyzed using IBM SPSS Statistics for Mac, version 27.0 (IBM Corp, Armonk, NY, USA).

### Ethical approval

The study was performed in accordance with the Declaration of Helsinki and approved by the Swedish Ethical Review Authority, which waived the need for written consent. Participants received written information about the study’s purpose and voluntary participation. Completion of questionnaire was considered as implied informed consent. Reference number: 2019−02368, with amendment 2021-06284-02.

## Results

A total of 1040 individuals responded to the survey, of whom 202 were excluded for completing only the background questions. The final sample comprised 838 participants, included in the analysis. The exact number of respondents is reported for each question. Information on the number of employees was available for 25 units. For these units, the median response rates (%; range) was 44 (8–92) for midwives, 20 (5–41) for pediatric physicians, and 13 (2–40) for obstetric physicians. Respondents included all major perinatal professional groups, with midwives as the largest. There was an even age distribution and clinical experience varied widely. Participation covered all major Swedish healthcare regions, with somewhat lower representation from the northern and southeastern areas. All NICU levels were represented, and overall, 40 of 44 labor units participated. Detailed characteristics are presented in Table 1.

**Table 1:** Background characteristic.

Variable (n)	N (%)	*N (%)*
**Occupation and education level (838)**		
Obstetric physician	114 (13.6)	
Obstetrician		*83 (72.8)*
Obstetric resident		*31 (27.2)*
Pediatric physician	121 (14.4)	
Neonatologist or Neonatal fellow		*41 (33.9)*
Pediatrician		*44 (36.4)*
Pediatric resident		*36 (29.8)*
Midwife	368 (43.9)	
Assistant nurse delivery ward	89 (10.6)	
Nurse	109 (13.0)	
Nurse specialized in pediatric care		*75 (68.8)*
General nurse		*34 (31.2)*
Assistant nurse neonatal ward	37 (4.4)	
**Age (824)**		
20–35 years of age	254 (30.8)	
36–50 years of age	349 (42.4)	
51–65 years of age	207 (24.7)	
> 65 years of age	14 (1.7)	
**Years of working experience (758)**		
0–5 years	269 (35.5)	
6–10 years	151 (19.9)	
11–20 years	174 (20.8)	
> 20 years	164 (19.6)	
**NICU level**^1^ **(838)**		
Level 1	94 (11.2)	
Level 2	500 (59.7)	
Level 3	244 (29.1)	
**Region of practice (838)**		
Northern region	45 (5.4)	
Middle region	216 (25.8)	
Eastern region	154 (18.4)	
Southeast region	57 (6.8)	
Western region	105 (12.5)	
Southern region	261 (31.1)	
**SAVE-study unit employee**	335 (40.0)	

Characteristics on survey participants (n = 838), presented as frequencies and percentages. Regions are divided according to the Swedish Association of Local Authorities and Regions.

^1^Neonatal intensive care unit (NICU) levels in Sweden.

Level 1: No neonatal intensive care; 8 units, corresponding to US level I.

Level 2: Providing partial neonatal intensive care; 24 units, corresponding to US level II-IIIA. Level 3: Providing full neonatal intensive care; 8 units, corresponding to US level IIIB-IV.

### Perceived importance of cord clamping timing

**Fig 1:**
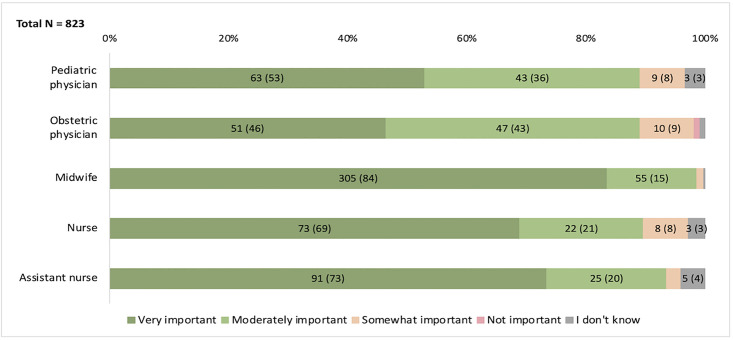
Responses on importance of cord clamping timing. Bar chart displaying responses from each professional group (pediatric- and obstetric physicians, midwives, nurses, and assistant nurses; y-axis). Bars are divided into colored segments representing response categories, with color explanations provided within the figure. N (%) are shown within bars where space permits. Complete data for all categories are provided in S1 Table. The total number of respondents for each profession is indicated on the y-axis.

Among included respondents, 94% (775/824), reported timing of CC being either “very important” or “moderately important” for neonatal outcomes. A statistically significant difference in responses was observed between the professional groups (Kruskal-Wallis test, p < 0.001). Post hoc pairwise comparisons showed that all nursing professions differed significantly from both physician groups (Mann-Whitney, p < 0.001). The overall response distributions for each group are presented in Fig 1.

**Fig 2:**
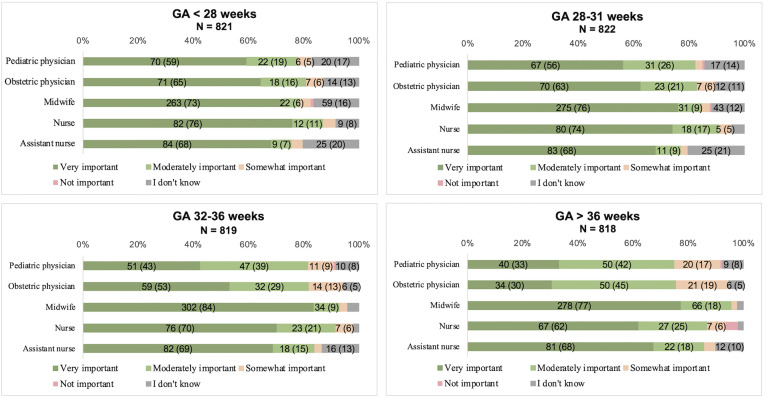
Responses on importance of cord clamping timing, stratified by gestational age. Bar charts display responses for each professional group (pediatric- and obstetric physicians, midwives, nurses, and assistant nurses; y-axis) across four gestational age groups: < 28 weeks, 28-31 weeks, 32-36 weeks, and > 36 weeks. Bars are divided into colored segments representing response categories, with color explanations shown in the figure. N (%) are shown within bars where space permits. Complete data for all categories are provided in S1 Table. The total number of respondents for each professional group is indicated on the y-axis of each chart.

When responses were analyzed by gestational age category, significant differences between nursing staff and physicians remained across all categories (Kruskal-Wallis, p < 0.005). The largest differences (post hoc Mann-Whitney, p < 0.001) were seen at higher gestational ages (> 36 weeks), while at lower gestational ages, significant differences remained primarily between midwives and physicians (p ≤ 0.005). Among physicians, there was a trend towards fewer respondents rating “very important” for higher gestational age categories, a pattern that was not observed among the nursing staff. The response alternative “I don’t know” was more frequently reported at lower gestational ages across all professions. Response distributions by gestational age category for each profession are presented in Fig 2.

### Timing of cord clamping

All responses stratified by profession and clinical situation are presented in Table 2. Most respondents preferred to clamp the cord ≥ 60 seconds or at cessation of umbilical pulsation. For normal vaginal births, 89% of midwives (312/351), preferred an event-based approach to CC rather than a time-based approach. Midwives and pediatric physicians differed in their answers on timing of CC in instrumental deliveries. Midwives were more inclined to use an event-based approach compared to pediatric physicians, 86% (298/346) vs 28% (17/60), respectively (Fisher’s exact test, p < 0.001). In elective cesarean sections, 71% of obstetric physicians, (74/104), considered a time-based approach to be most suitable, with the predominant response being “60 seconds to 3 minutes”.

**Table 2:** Approach to timing of cord clamping.

			Time based approach to cord clamping, n (%)					Event based approach to cord clamping, n (%)			
			< 30 s	30 - 60 s	60 s – 3 min	3 - 6 min	> 6 min	When pulsations cease	At placental delivery	When pulsations ceases or at placental delivery	Total event based
**Vaginal birth**											
**Normal**											
Midwife, n = 351			0 (0.0)	1 (0.3)	8 (2.3)	24 (6.8)	6 (1.7)	**188 (53.6)**	88 (25.1)	36 (10.3)	312 (88.9)
**Instrumental**											
Midwife, n = 346			0 (0.0)	4 (1.2)	11 (3.2)^3^	28 (8.1)^2^	5 (1.4)	**191 (55.2)** ^**3**^	75 (21.7)^3^	32 (9.2)	298 (86.1)^3^
Pediatric physician, n = 60			1 (1.7)	2 (3.3)	**26 (43.3)** ^**3**^	13 (21.7)^2^	1 (1.7)	16 (26.7)^3^	0 (0.0)^3^	1 (1.7)	17 (28.3)^3^
**Cesarean section**											
**Elective**											
Obstetric physician, n = 104			1 (1.0)	11 (10.6)	**60 (57.7)**	2 (1.9)	0 (0.0)	24 (23.1)	5 (4.8)	1 (1.0)	30 (28.8)
**Emergency (maternal indication)**											
Obstetric physician, n = 106			6 (5.7)	16 (15.1)	**56 (52.8)**	3 (2.8)	0 (0.0)	22 (20.8)	3 (2.8)	0 (0.0)	25 (23.6)
**Emergency (fetal indication)**											
Obstetric physician, n = 104			20 (19.2)	31 (29.8)	**35 (33.7)**	3 (2.9)	0 (0.0)	12 (11.5)	3 (2.9)	0 (0.0)	15 (14.4)
**Resuscitation**											
**Vaginal**											
Midwife, n = 326			45 (13.8)	**113 (34.7)**	51 (15.6)	21 (6.4)	9 (2.8)	66 (20.2)^1^	19 (5.8)	2 (0.6)	87 (26.7)^2^
Pediatric physician, n = 59			13 (22.0)	**28 (47.5)**	8 (13.6)	4 (6.8)	0 (0.0)	5 (8.5)^1^	1 (1.7)	0 (0.0)	6 (10.2)^2^
**Cesarean**											
Obstetric physician, n = 112			**79 (70.5)** ^**3**^	23 (20.5)^2^	3 (2.7)^1^	0 (0.0)	1 (0.9)	4 (3.6)	2 (1.8)	0 (0.0)	6 (5.4)
Pediatric physician, n = 61			18 (29.5)^3^	**27 (44.3)** ^**2**^	8 (13.1)^1^	2 (3.3)	0 (0.0)	5 (8.2)	1 (1.6)	0 (0.0)	6 (9.8)

Timing of cord clamping among different professional groups by mode of delivery and in resuscitation situations. Response options are presented in columns and represent time-based responses (blue shading) and event-based responses (orange shading). Modal responses for each profession and delivery scenario are indicated in bold. Where professional groups were asked about the same delivery scenario, responses were compared. P-values are indicated in footnotes: ¹ = p < 0.05, ² = p < 0.01, ³ = p < 0.001.

#### Resuscitation.

Attitudes toward CC in resuscitation situations varied by profession (Table 2). Most obstetric physicians (71%, 79/112) preferred CC < 30 seconds, compared to 30% (18/61) of pediatric physicians, who more often chose 30–60 seconds (Fisher’s exact test, p < 0.001). For vaginal births requiring resuscitation, midwives were more prone to choose a time-based approach, though 27% (87/326) still preferred event-based CC, versus 10% (6/59) of pediatric physicians. The proportion of midwives preferring event-based CC during resuscitation did not differ significantly between SAVE and non-SAVE units, 31% (34/109) vs 24% (53/217), respectively (Fisher’s exact test, p = 0.23).

Regarding delayed CC (>1 min) during resuscitation, 47% (53/113) of obstetric and 46% (30/65) of pediatric physicians agreed it should be avoided, while 51% (184/360) of midwives disagreed.

### Clinical situations affecting time to cord clamping

Both midwives and obstetric physicians were inclined to clamp immediately in certain situations: for maternal postpartum hemorrhage, 78% (282/363) of midwives and 89% (100/112) of obstetric physicians; for potential delays in neonatal resuscitation, 88% (317/362) and 92% (105/114), respectively. Regarding risk of neonatal jaundice, “no effect on my practice” was reported by 67% (241/362) of midwives and 44% (50/114) of obstetric physicians, followed by “no opinion” (12% (42/362) and 27% (31/114), respectively. Responses concerning neonatal hypothermia were similar, with 54% (197/363) of midwives and 41% (47/114) of obstetric physicians indicating no effect on their practice. Among midwives, 60% considered parental preferences regarding CC time to be very important, while only 3% regarded it as not important at all.

### Cord clamping guidelines

Approximately 60% (504/815) of health care professionals reported a local written guideline recommending delayed CC for vigorous neonates born vaginally, and 42% (353/817) for cesarean deliveries. “I don’t know”, was the second most common response (24% (203/815) for vaginal births, 34% (287/817) for cesarean sections). For guidelines on CC management in neonates needing resuscitation, “I don’t know” was predominant (35% (295/810) for vaginally born, 40% (333/812) for cesarean sections). Assistant and pediatric nurses more often responded “I don’t know” than midwives and obstetricians across all scenarios (Pearson chi-square, p < 0.001). Pairwise Fisher’s exact test with Bonferroni correction confirmed significant differences compared to midwives in all scenarios, and to obstetricians for cesarean sections.

## Discussion

This cross-sectional survey revealed that health care professionals involved in neonatal resuscitation considers timing of umbilical CC being an important issue. This result was somewhat expected since Sweden has a long tradition of delayed CC in vigorous neonates born vaginally, with recommended time to CC > 2 minutes since 2008 [25]. Recent observational studies have identified a median CC time of 6 minutes for vigorous neonates born vaginally [26,27]. The general agreement on the importance of CC timing suggests high acceptability – a key implementation outcome according to Proctor et al. - which is important for successful adoption of new clinical practices [23]. However, variations were observed in health care professionals’ attitudes regarding the significance of CC timing, as well as in their preferences for time-based versus event-based approach.

Our analysis revealed variations between professions, with midwives categorizing timing of CC as “very important” to a greater extent than obstetricians and pediatricians. Midwives were also more likely to employ an event-based approach for determining CC timing. This is in line with international and national research. An integrative review by Peberdy et al. identified that attitudes and opinions about CC timing differed among midwives and obstetricians, where midwives preferred delayed CC to a higher degree [28]. However, while most obstetric and pediatric physicians preferred a time-based approach for CC, unexpectedly many physicians considered “cessation of pulsations” as the most appropriate criterion for timing in elective cesarean sections and instrumental deliveries. The attitude towards extended CC times observed among Swedish physicians appears to differ from that reported in studies from other countries. In a Dutch survey from 2015 by Boere et al. early CC < 1 minute was applied in 81% of the obstetric practices in cesarean sections [29]. In a study by Leslie et al from 2018, only 3.0% of U.S. obstetricians indicated a preference for delaying CC until pulsation cessation in healthy, term neonates delivered by cesarean section [18]. In a more recent study from 2021 by Aydogan Kirmizi et al. 75.8% of Turkish obstetricians stated that they clamp the cord within the first minute and only 2.3% waited until pulsations ceased [30]. While our study does not provide data on reasons for this preference, the high proportion of obstetricians selecting delayed CC until cessation of pulsations during cesarean section may possibly reflect the influence of the Swedish midwife-led care model and a professional culture that emphasizes physiological birth and interdisciplinary collaboration [31]. However, this interpretation remains speculative.

Although there seemed to be some consensus among midwives and pediatric physicians regarding timing of CC in resuscitation situations, a surprisingly high share of midwives preferred waiting until cessation of pulsations or until placenta was delivered. It is possible that midwives selecting this option might have presumed an ICR-setting with mobile equipment; however, we did not collect data on this aspect, and this interpretation remains speculative. It may also reflect that midwives historically have been more prone to clamp the cord only after the onset of respiration or delivery of the placenta [32]. The lack of difference between midwives working in SAVE and non-SAVE units may suggest a broader acceptance of ICR and longer clamping times in Sweden, but this interpretation should be made cautiously given the study’s limitations. For neonates requiring resuscitation after cesarean delivery, obstetric physicians preferred CC within 30 seconds of birth, earlier than what the pediatric physicians stated. This variability in timing between physicians could perhaps be due to time pressure to transfer the neonate to the neonatal team, a barrier to delayed CC identified among midwives [33]. Both midwives and physicians cared to avoid delay of resuscitation, but a stronger reluctance for early CC was identified among midwives. Although the research on ICR remains limited, the results align with other studies indicating a strong preference among midwives for delayed CC, even in situations requiring interventions [34–36]. This inclination parallels recent research advocating for a shift from time-based CC toward a physiological-based approach [37]. Another notable finding was the frequent “I don’t know” responses about local guidelines, particularly in resuscitation scenarios and among staff less involved in CC decisions, likely reflecting professional roles. In Sweden, national guidelines on CC timing are clear for most deliveries, possibly contributing to generally liberal attitudes. However, for resuscitation, national guidelines are less explicit, and as local protocols often follow these, clarity may also be lacking locally. This may explain the higher “I don’t know” responses rate and emphasize the need for clear cord management protocols during resuscitation.

In summary, our findings show a general consensus regarding the importance of CC timing and a relatively high proportion of physicians favoring event-based clamping, but at the same time reveal differences between professional groups and gaps in institutional guidance, highlighting challenges to achieving consistent practice. According to Proctor et al., acceptability is influenced not only by individual attitudes but also by organizational context and support [23]. Addressing both these dimensions is essential to enhance acceptability and thereby facilitate successful implementation of evidence-based guidelines.

### Strengths and limitations

This study has several strengths and limitations. This is, to our knowledge, the only nationwide study investigating attitudes towards umbilical CC including the three professional categories midwives, obstetric physicians, and pediatric physicians. The broad geographic distribution and representation from all NICU levels and nearly all labor units, support the relevance of the findings. The somewhat lower response rates from the northern and southeastern regions may be related to the smaller population size in these areas [38]. The study covers attitudes towards CC timing in different settings, from normal births to resuscitation situations and in both vaginal births and cesarean sections. The survey was derived from a previously used questionnaire [24]. This facilitates an understanding of attitudes towards CC timing for comparison across different countries and settings.

The questions in the survey regarding the timing of CC were directed to different contexts depending on profession. This gives credibility to the responses but also limits the possibility to compare answers between different professions. The total response rate in the study is unknown. Unit-level response rates were estimated using staff numbers obtained from a separate survey distributed concurrently with the main survey. These figures may be imprecise, and thus the true response rate remains uncertain and the potential for nonresponse bias cannot be excluded. Moreover, the generalizability of the findings is limited by these methodological constraints and by institutional differences. However, the coherent response within each profession strengthens the credibility of the results. As with all voluntary, self-reported surveys, our findings may also be influenced by the respondents’ tendency to provide socially desirable answers, and since the survey was voluntary there is a potential for self-selection bias. Nevertheless, despite these limitations, we believe this study will add valuable information about attitudes toward CC timing in the multidisciplinary team. As described by Anton et al. improvement of multidisciplinary collaboration is an important part of overcoming barriers to implementation [22].

## Conclusion

The study reveals a generally positive attitude towards delayed CC among health care professionals in Sweden, but notable differences exist between professional groups, particularly in resuscitation situations. These findings highlight gaps in institutional guidance and underscore the need for harmonized, interdisciplinary protocols to ensure consistent practice. The overall positive attitudes suggest a favorable environment for implementing ICR in Sweden. Our results may inform the development of clinical guidelines and targeted training initiatives to support this process. Further research is needed to evaluate the practical challenges and outcomes of ICR implementation.

## Supporting information

S1 Appendix Study questionnaire. The full questionnaire used in the present study, including all items and response options.
(PDF)

S1 Table Complete numerical data for Figs 1 and 2. This table presents all underlying numerical data for Figs 1 and 2, including all response categories and values, also those not displayed in the figure bar segments.
(PDF)
