# Supplementary material for: Attitudes and beliefs regarding umbilical cord clamping among midwives, obstetricians, and neonatologists in Sweden: A national cross-sectional survey
Source: PLoS One. 2025 Oct 8;20(10):e0332745. doi: 10.1371/journal.pone.0332745 (PMC12507212; doi:10.1371/journal.pone.0332745)
Supplement: S1 Appendix — The full questionnaire used in the present study, including all items and response options. (PDF) [file pone.0332745.s001.pdf]

Questionnaire "Attitude towards cord clamping"

1. How important is the time of umbilical cord clamping at birth, according to you?

Very important	Moderately important	Somewhat important	Not important at all	I don't know

2. How important do you think the time of umbilical cord clamping is for neonatal outcomes at the following gestational ages?

**Gestational week**	Very important	Moderately important	Somewhat important	Not important at all	I don't know
<28 weeks					
28–31 weeks					
32–36 weeks					
>36 weeks					

3. Do you know if there is a written guideline for cord clamping at your clinic…

a) … for vigorous neonates born vaginally?

Yes, there is a guideline for delayed cord clamping (2-3 min)	
Yes, there is a guideline for early cord clamping	
No, there is no guideline	
I don't know	

b) …for neonates in need of neonatal resuscitation after vaginal birth?

Yes, there is a guideline for delayed cord clamping	
Yes, there is a guideline for early cord clamping	
No, there is no guideline	
I don't know	

c) … for vigorous neonates born by cesarean section?

Yes, there is a guideline for delayed cord clamping (≥ 1 min)	
Yes, there is a guideline for early cord clamping	
No, there is no guideline	
I don't know	

d)…for neonates in need of neonatal resuscitation after cesarean section?

Yes, there is a guideline for delayed cord clamping	
Yes, there is a guideline for early cord clamping	
No, there is no guideline	
I don't know	

Questionnaire "Attitude towards cord clamping"

4. Please indicate what you think the timing of umbilical cord clamping at each of the following situations should be in term neonates (≥ 37 gestational weeks):

	Time guided					Clinically guided	
	< 30sec	30-60 sec	> 60sec – 3 min	> 3 – 6 min	> 6 min	Until pulsations cease	When the placenta delivers
Normal Vaginal birth							
Instrumental vaginal delivery (vacuum extraction, forceps)							
Vaginal birth when there is a need for neonatal resuscitation							
Elective cesarean							
Emergency cesarean on maternal indication							
Emergency cesarean on fetal indication							
Emergency cesarean when there is a need of neonatal resuscitation							

5. Please indicate how much you agree or disagree with the following statements regarding the timing of umbilical cord clamping for term neonates (≥ 37 gestational weeks):

a) vaginal birth

	Strongly/ mostly disagree	Somewhat disagree	Neither agree nor disagree	Somewhat agree	Strongly/ mostly agree
Cord clamping (> 1 min) should not be delayed if immediate resuscitation is indicated.					
Umbilical cord milking can be used in place of delayed cord clamping.					

b) cesarean section

	Strongly/ mostly disagree	Somewhat disagree	Neither agree nor disagree	Somewhat agree	Strongly/ mostly agree
Cord clamping (> 1 min) should not be delayed if immediate resuscitation is indicated.					
Umbilical cord milking can be used in place of delayed cord clamping.					

6. Please indicate the extent to which each of the following potential morbidities affects your umbilical cord clamping recommendations or practice in term infants (≥37 gestational age):

	Makes me very inclined to delay	Makes me somewhat inclined to delay	Has no effect on my practice	Makes me somewhat inclined to clamp immediately	Makes me very inclined to clamp immediately	I have no opinion/ I don't know
**Maternal**						
Maternal hemorrhage						
**Neonatal:**						
Hyperbilirubinemia						
Hypothermia						
Delay in resuscitation						

7. How important are the parents' wishes for time to cord clamping for your handling of the birth?

Very important	Moderately important	Somewhat important	Not important at all	I don't know

---

Inspired by Jelin AC, Kuppermann M, Erickson K, Clyman R, Schulkin J. Obstetricians' attitudes and beliefs regarding umbilical cord clamping. The Journal of Maternal-Fetal & Neonatal Medicine. 2014;27:1457-61.
