# Supplementary material for: Attitudes and beliefs regarding umbilical cord clamping among midwives, obstetricians, and neonatologists in Sweden: A national cross-sectional survey
Source: PLoS One. 2025 Oct 8;20(10):e0332745. doi: 10.1371/journal.pone.0332745 (PMC12507212; doi:10.1371/journal.pone.0332745)
Supplement: S1 Table — This table presents all underlying numerical data for Figs 1 and 2, including all response categories and values, also those not displayed in the figure bar segments. (PDF) [file pone.0332745.s002.pdf]

Gestational age/Profession	Very important N (%)	Moderately important N (%)	Somewhat important N (%)	Not important at all N (%)	I don't know N (%)
**Gestational age not specified**					
Pediatric physician	63 (53)	43 (36)	9 (8)	0 (0)	3 (3)
Obstetric physician	51 (46)	47 (43)	10 (9)	1 (< 1)	1 (< 1)
Midwife	305 (84)	55 (15)	4 (1)	0 (0)	1 (< 1)
Nurse	73 (69)	22 (21)	8 (8)	0 (0)	3 (3)
Assistent nurse	91 (73)	25 (20)	3 (2)	0 (0)	5 (4)
**< 28 weeks**					
Pediatric physician	70 (59)	22 (19)	6 (5)	0 (0)	20 (17)
Obstetric physician	71 (65)	18 (16)	7 (6)	0 (0)	14 (13)
Midwife	263 (73)	22(6)	14 (4)	4 (1)	59 (16)
Nurse	82 (76)	12 (11)	5 (5)	0 (0)	9 (8)
Assistent nurse	84 (68)	9 (7)	5 (4)	0 (0)	25 (20)
**28-31 weeks**					
Pediatric physician	67 (56)	31 (26)	3 (3)	1 (< 1)	17 (14)
Obstetric physician	70 (63)	23 (21)	7 (6)	0 (0)	12 (11)
Midwife	275 (76)	31 (9)	10 (3)	2 (< 1)	43 (12)
Nurse	80 (74)	18 (17)	5 (5)	0 (0)	5 (5)
Assistent nurse	83 (68)	11 (9)	3 (2)	0 (0)	25 (21)
**32-36 weeks**					
Pediatric physician	51 (43)	47 (39)	11 (9)	1 (< 1)	10 (8)
Obstetric physician	59 (53)	32 (29)	14 (13)	0 (0)	6 (5)
Midwife	302 (84)	34 (9)	10 (3)	0 (0)	15 (4)
Nurse	76 (70)	23 (21)	7 (6)	0 (0)	2 (2)
Assistent nurse	82 (69)	18 (15)	3 (3)	0 (0)	16 (13)
**> 36 weeks**					
Pediatric physician	40 (33)	50 (42)	20 (17)	1 (< 1)	9 (8)
Obstetric physician	34 (30)	50 (45)	21 (19)	0 (0)	6 (5)
Midwife	278 (77)	66 (18)	7 (2)	0 (0)	8 (2)
Nurse	67 (62)	27 (25)	7 (6)	5 (5)	2 (2)
Assistent nurse	81 (68)	22 (18)	5 (4)	0 (0)	12 (10)
